# Rapid and Sensitive Method for Extraction of *Plicosepalus acacia* with Determination of Its Main Polyphenolic Compounds Using Validated HPLC

**DOI:** 10.1155/2020/9598606

**Published:** 2020-07-24

**Authors:** Mashail N. AlZain, Rashed N. Herqash, Abdulaziz N. Almoqbil, Omer Mohammed Almarfadi, Mansour N. Ibrahim, Riaz Ullah, Omar M. Noman

**Affiliations:** ^1^Department of Biology, College of Sciences, Princess Nourah Bint Abdulrahman University, Riyadh 11451, Saudi Arabia; ^2^Medicinal, Aromatic, and Poisonous Plants Research Center, College of Pharmacy, King Saud University, Riyadh, Saudi Arabia; ^3^Department of Pharmacognosy, College of Pharmacy, King Saud University, P.O. Box 2457, Riyadh 11451, Saudi Arabia; ^4^Department of Pharmacognosy, College of Pharmacy, Aden University, P.O.Box 6312, Aden, Yemen; ^5^Department of Agricultural Engineering, College of Food and Agriculture Sciences, King Saud University, Riyadh 11451, Saudi Arabia

## Abstract

Matrix solid phase dispersion (MSPD) trailed by HPLC is a quick and fruitful technique utilized for fortitude of flavonoids such as Catechin, Kaempferol, Quercetin, and Rutin existing in *P*. *acacia*. The trial parameters that influenced the extraction potential (comprising the mass ratio of sample to the dispersant, nature of dispersant, and the nature of elution solvent and its volume) were examined and optimized. These MSPD optimized parameters regulated are as follows: 8 mL of methanol was utilized as elution solvent, silica gel/sample mass ration was selected to be 2 : 1, and dispersing sorbent was silica gel. The technique retrievals were regulated to be “from 96.87 to 100.54%” and the RSDs from 1.24 to 4.45%. The product of extract obtained by MSPD method was larger than that of other methods, i.e., sonication extraction or traditional reflux with lessened necessities on time, sample, and solvent.

## 1. Introduction

Flavonoids are a significant class of regular items; particularly, they have a place with a class of plant auxiliary metabolites with a polyphenolic structure, ordinarily found in natural products, vegetables, and many drinks. They have various helpful cell reinforcement and biochemical impacts related to different ailments, for example, Alzheimer's infection (AD), cancer, and atherosclerosis [1–3]. Flavonoids are related to a wide scope in health issues and are a fundamental part in various restorative, therapeutic, nutritional, and nutraceutical applications due to their anticarcinogenic, antimutagenic, anti-inflammatory, and antioxidant potential as well as their capacity of adjusting basic cell catalyst capacities. It is also measured to be powerful enzyme inhibitors, such as phosphoinositide 3-kinase, lipoxygenase, xanthine oxidase (XO), and cyclooxygenase (COX) [4–6]. Modern drifts in the world lead to advancement in flavonoid innovative work, isolation/characterization, and recognition as well as functions and medical utilization of flavonoids and its outcomes. The Kingdom of Saudi Arabia comprehends eighty percent of the Arabian Peninsula with greater than twelve hundred plant species having important medicinal uses and diversity [7]. The unfriendly parameters of environment such as low supplement soils, shortage of water, and extremely high temperatures in the Kingdom of Saudi Arabia are measured as encouraging highlights for therapeutic plants which stretch them with more chemical defense compared to the plants grown in the best environmental conditions [8]. The indigenous plants of KSA are considered rich source of flavonoid content due to harsh environmental conditions. *P*. *acacia* specie belonging to Loranthaceae family is throughout distributed in the Kingdom of Saudi Arabia which has testified to have numerous flavonoid agents including Kaempferol, Catechin, Quercetin, and Rutin [9, 10]. Because of the wide scope of natural exercises of flavonoids content, distribution of these constituents has expanded overwhelmingly and picked up fame. Yet, still the yield and scattering of these constituents are disturbed by diverse plant heritages and reap periods. Consequently, simple, reliable, and fast techniques are highly needed for the extraction and major flavonoids fortitude in *P*. *acacia*. In 1989, Barker presented the innovation of solid phase dispersion matrix (MSPD) as an appealing technique to extricate medical entity from the tissue of animals [11]. From that point forward, the procedure has pulled in extensive enthusiasm as it joins interruption, extraction, and cleaning in one stage and successfully separates the semisolid, solid, and extremely sticky samples [12]. The exceptional goods of MSPD give quick, minimal effort and helpful favorable circumstances contrasted with conventional techniques. Effective utilization of MSPD to take care of numerous troublesome scientific issues has demonstrated its extraordinary potential to diminish specialist time, increment test profitability, abbreviate time, and limit dissolvable use and related expenses for the acquirement and removal of solvents, just as giving systematic outcomes that are equivalent to or better than old style or authority techniques [13, 14]. MSPD has of late been utilized as a potential and compelling alternative to customary extraction techniques for extricating constituents from therapeutic plants [12–16]. However, there is no examination on MSPD as a type of extraction of flavonoids from *P*. *acacia* in the available literature as far as we could possibly know. In the present investigation, MSPD was utilized as an elective example planning technique followed by HPLC separation for the extraction and assurance of four flavonoids in *P*. *acacia* for the purpose of getting maximum amount of yield. The properties of the altered strategic factors comprising elution solvent volume, elution solvent, dispersing sorbent, and dispersing ratio of sorbent to sample on the MSPD extraction competence of flavonoids have been broadly assessed and optimized. A comprehensive authentication for the HPLC technique was then estimated, comprised of quantification, detection limits, accuracy, precision, and linearity. Furthermore, the extraction yields produced by the MSPD were matched with those achieved by customary ultrasonic and Soxhlet techniques. Zhang et al. method of extraction was followed and adopted [17].

## 2. Materials and Methods

### 2.1. Chemicals and Reagents

**Figure 1:**
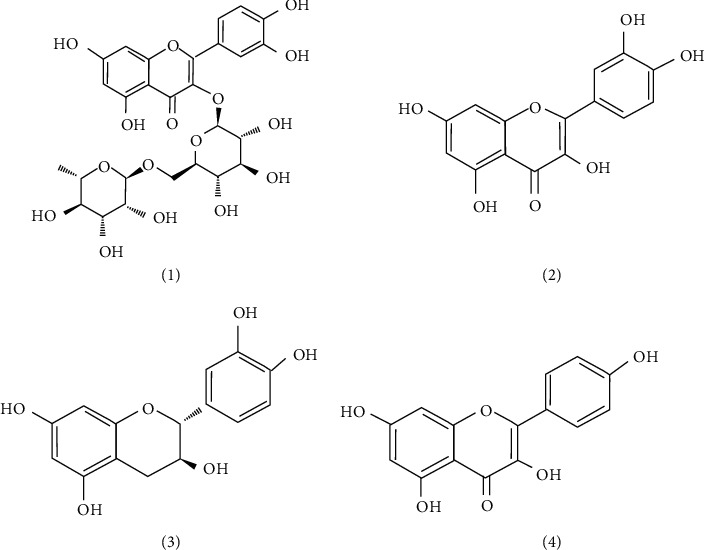
The chemical structures of the four phenolic compounds.

The Rutin (1), Quercetin (2), Catechin (3), and Kaempferol (4) are selected as standards. The organic structures of these four constituents are shown in Figure 1.

### 2.2. Plant Material

The leaves of medicinal plant *P*. *acacia* were collected from the south of KSA in March 2012. Five samples of grinded aerial part (100 g) were dried at a constant temperature of 60°C for 24 h and filtered through an 80-mesh sieve.

### 2.3. Apparatus and Operating Parameters

Shimadzu chromatographic system (Shimadzu, Kyoto, Japan) was used for LC analysis equipped with two LC-10AD Prominence liquid chromatography pumps, SPD-20A Prominence UV detector (set at 210 nm), and System controller CBM-20 A/20Alite with Lab solutions (LC solution) software. Separation was performed with a Shim-pack XR ODS column 150 × 4.6 mm, 5 *μ*m particle size (Shimadzu, Kyoto, Japan) by gradient elution. Acidified water (5% acetic acid, v/v) and acetonitrile were used as mobile phases A and B, respectively. The gradient was programmed as follows: 0–10 min, 0–30% B; 10–25 min, 30–85% B; 25–30 min, 85–100% B; 30–35 min, 100% B. The analysis was performed at constant flow rate of 0.8 ml/min and an injection volume of 20 L. The compounds were quantified with external standards, using calibration curves.

### 2.4. MSPD Extraction Method

A powder of 0.1 g of *P*. *acacia* sample and 0.2 g of silica gel was put in an agate mixer and gently mixed together for 5 minutes to achieve a homogeneous mixture. After the *P*. *acacia* sample was fully dispersed and disrupted, the mixture was inserted into a 5 mL glass syringe prefilled with a layer of adsorbent cotton at the bottom of the syringe. Then, another layer of adsorbent cotton was added to the top of the sample mixture by mild compression using a syringe plunger to remove unwanted channels. The sealed syringe was eventually rinsed with 4 mL of methanol. The eluent was collected in a 10 mL of volumetric flask and made up to 10 ml with MeOH. The obtained solution was filtered through a 0.45 *µ*m filter and the resulting solution was analyzed by HPLC.

### 2.5. Soxhlet Extraction Method

Soxhlet extraction method was used since old time and is still studied as the primary source for effective extraction [17]. Approximately 0.5 g of *P*. *acacia* sample was employed in a cellulose thimble holder and positioned in a Soxhlet extractor. Then, 90 mL MeOH was added into distillation flask and heated under reflux for 6 h. After an extraction course, the extract was moved into a 100 mL of volumetric flask and threw up to the mark with CH3OH. The ensuing extract solution was sieved through 0.45 *µ*m filter and the resulting solution was analyzed by HPLC.

### 2.6. Sonication Extraction Method

This method of sonication extraction was documented as an official method in Chinese Pharmacopoeia. Exactly 0.25 g of *P*. *acacia* sample was retained into a 20 mL of volumetric flask; then we added 18 mL of MeOH as the extraction solvent. The sonication course of extraction was stirred for 20 min. Then, the sample solution was attuned to the streak with CH_3_OH. The sample solution was filtered through a 0.45 *µ*m filter and the resulting solution was analyzed by HPLC.

## 3. Results and Discussion

### 3.1. Optimization of MSPD Procedure

As we all know, MSPD is an efficient extraction method that could combine destruction, extraction, and cleaning functions in one step. So as to accomplish the most effective extraction harvests of flavonoids from *P*. *acacia*, the most appropriate extraction factors for MSPD strategy were considered. The analyses were executed to streamline the initial factors such as proportion of scattering sorbent to sample, sort of scattering sorbent, and volume of the eluting dissolvable and its nature, which decide the extraction yield of final concentrate.

#### 3.1.1. Selection of Dispersing Sorbent

**Figure 2:**
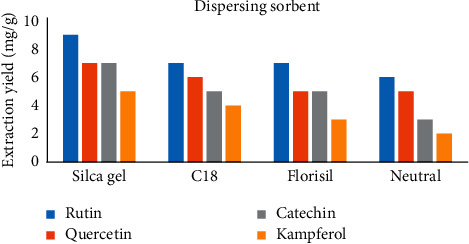
The effect of the dispersing sorbents on extraction yields.

Sorbent dispersion serves as both bound and rough solvent that breaks the sample matrix, scatters its constituents, and increases effective solvent-sample interaction during the MSPD mixing process. The specific selectivity of an MSPD procedure is therefore highly dependent on the sorbent used [33]. Four continual dispersing sorbents, comprising Florisil, C18, silica gel, and neutral alumina, were tried in this phase.  Figure 2 shows flavonoid extraction yields from *P*. *acacia* obtained from the four separate dispersing sorbents. It was clear that the lowest yields were produced by the extraction with neutral alumina. As can be seen, the yields of extraction of four flavonoids were slightly higher when using silica gel than the yields of extraction with Florisil and C18. Hence, silica gel was the scattering sorbent designated for MSPD technique as it furnished the finest extraction production of the four flavonoids and was comparatively low in cost value.

#### 3.1.2. Effect of the Sample/Sorbent Mass Ratio

**Figure 3:**
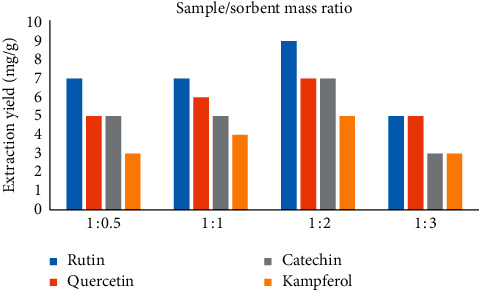
Mass ratio of sample to silica.

A reasonable proportion of scattering sorbent to sample could build the contact zone among test and scattering sorbent and advance the adsorption of investigations on scattering sorbent. Hence, four different sample-to-silica gel mass ratios ranging from 0.5 : 1 to 1 : 3 were evaluated. The investigational results are displayed in Figure 3. It was exposed that the capacity of yield of extraction increases with the increase in sample-to-silica gel mass ratios and the sample-to-silica gel mass ratio at 1 : 2 resulted in the highest extraction yield for four flavonoids. However, a further increase in the mass ratio of 1 : 3 resulted in a reduction in the extraction yields of four flavonoids, which was likely due to the strong silica gel absorbability. Thus, in this work, the optimized mass ratio was chosen at 1 : 2.

#### 3.1.3. Effect of Elution Solvents

**Figure 4:**
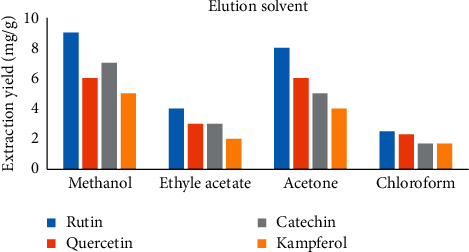
The effect of elution solvents on extraction yields.

The nature of the elution solvent is also appropriate in the MSPD manner, as the marked constituent should be selectively desorbed while the other matrix interference components should be retained in the column. The elution solvent performs two important roles: the first is the separation in which the profile appears as a general mobile phase and the second is the dissolution/extraction of target compounds [18]. Consequently, four solvents with altered polarity were calculated to hand-pick the appropriate solvent for extraction of the flavonoids from *P*. *acacia*. The solvents used were methanol, ethyl acetate, acetone, and dichloromethane. The figures of these findings are mentioned in Figure 4. It can be clearly found out from the given figures that when methanol was utilized for the elution, all flavonoids revealed the uppermost extraction yields. Conversely, the use of chloroform as an elution solvent resulted in the lowest extraction yield of flavonoids. Generally, use of methanol afforded the highest yields extraction of all flavonoids tried and was thus utilized as the elution solvent for the MSPD extraction route.

#### 3.1.4. Effect of the Volume of Elution Solvent

**Figure 5:**
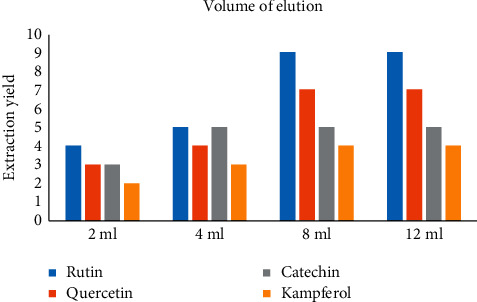
The effect of volume of elution on extraction yields.

When performing an MSPD extraction procedure, the elution volume is another essential factor that we desire to study. Experiments with four different volumes of methanol (2, 4, 8, and 12 mL) were investigated and results are illustrated in Figure 5. The results showed that extraction yields for four compounds increased slowly with adding methanol volume from 2.0 mL to 8.0 mL, and then the extraction yields were kept unchanging even further increasing the volume to 12.0 mL. Therefore, 8.0 mL of methanol was selected to ensure complete desorbing of the compounds from dispersing sorbent at the minimum solvent consumption.

### 3.2. Validation of the MSPD-HPLC Method

**Figure 6:**
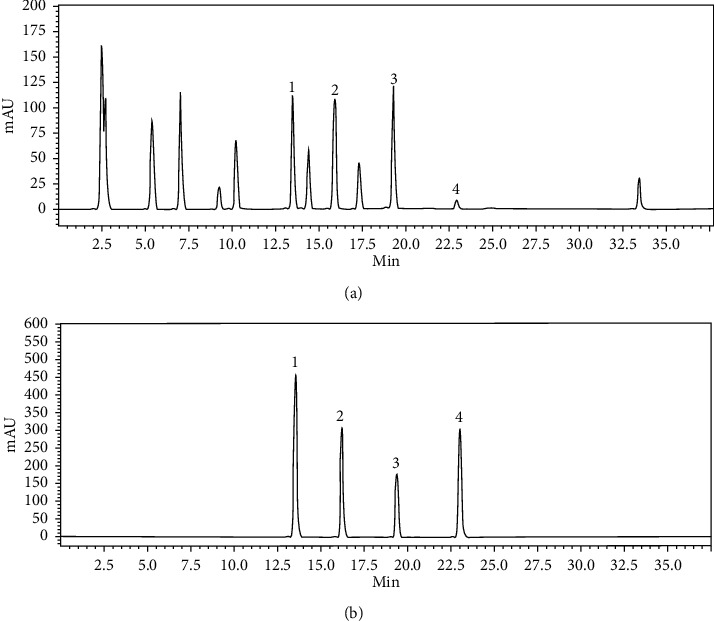
HPLC chromatograms of (a) extract and (b) standard flavonoid compounds. Peaks: (1) Rutin, (2) Quercetin, (3) Catechin, and (4) Kaempferol.

HPLC analysis chromatographic peaks were recognized by comparing the retention times of individual standards with the extract. HPLC chromatogram (Figure 6) of the extract showed sharp peaks for RU, QU, CA, and KA. Once the parameters that affect the MSPD procedure had been determined, the performance features of the proposed method (MSPD-HPLC) were evaluated by validation of the method with *P*. *acacia* samples according to International Conference on Harmonization (ICH) guidelines [19]. The proposed method was tested in terms of linearity, sensitivity, precision, and accuracy.

#### 3.2.1. Calibration Curves, Linearity, and Sensitivities (Detection Limits)

The linearity of the detector response was determined based on calibration curves. Linear calibration curves were constructed using six concentration points and plotting UV detector response in terms of peak area of individual four flavonoids (*y*-axis) versus the concentration (*x*-axis) of each injection for each flavonoids mixture and separate compound. All calibration curves showed good linearity correlations in the range *r*^2^ = 0.9996 − 0.9998. The limits of detection (LOD) and limit of quantification (LOQ) were used to evaluate the sensitivity of the method. LOD and LOQ for each detected analyte were calculated based on the calibration curve and calculated according to

(1) $$\begin{array}{c} \text{LOD}=3.3\frac{\sigma }{S}, \end{array}$$

(2) $$\begin{array}{c} \text{LOQ}=10\frac{\sigma }{S}, \end{array}$$

where *σ* is the standard deviation of the response and *S* is  the slope of the calibration curve. The LOD of four flavonoids ranged from 3.97 to 5.07 *µ*g/mL, and LOQ ranged from 12.05 to 15.39 *µ*g/m, respectively (Table 1). The “LODs and LOQs” acquired in this study are low appropriately to ensure satisfying analysis of these compounds in real samples.

#### 3.2.2. Precision

To confirm correct quantification, repeatability and intermediate precision of the proposed method were evaluated and expressed as relative standard deviation (RSD). Precision was assessed by replicate analysis (*n* = 6) of MSPD extraction sample within 1 day (intraday) and between three consecutive days (interday). The assay gave satisfactory results as RSD values were 0.97–1.68 and 1.65–2.47% for intra- and interassay precisions, respectively (Table 1).

#### 3.2.3. Accuracy

The accuracy of the proposed method was determined by means of a recovery experiment, adding known quantities of standard solutions in three concentration levels (80%, 100%, and 120%). The untreated *P*. *acacia* samples were fortified with the standard analyte stock solutions at three different concentrations according to the linearity range. Blank “unfortified” samples were previously analyzed to detect the presence of RU, QU, CA, and KA. Fortified samples were analyzed and the results were represented as percent recovery (%).  Table 2 shows that mean recoveries of the investigated compounds ranged from 96.87 to 100.54% and their% RSDs at each level concentration were all less than 4.45%, which indicated that the proposed method was accurate enough for the quantification of the four flavonoid compounds in *P*. *acacia*.

### 3.3. Comparison of MSPD, Soxhlet, and Sonication Procedure

Table 3 lists the comparison of relevant characteristics for MSPD, Soxhlet, and the method of sonication extraction. It was shown that the overall extraction yield of the four compounds obtained by the MSPD was a little higher than that of traditional methods of extraction of Soxhlet and sonication. In comparison, the MSPD approach needed just 0.1 g sample, 4 mL organic solvent, and 15 min to extract *P*. *acacia* target flavonoids. It revealed that sample, organic solvent, and time consumption were reduced by the MSPD approach compared to traditional extraction methods like Soxhlet and sonication and other new techniques. More importantly, the extraction of MSPD does not require heating; thus this mild extraction condition could avoid any possible loss and degradation of target compounds during the extraction process. Ultimately, in terms of instrumental criteria, MSPD is also beneficial. Implementing the method requires only a cheap cartridge and mortar that could be created by any chemical laboratory [13]. Ultimately, these benefits show that the extraction of MSPD from *P*. *acacia* should be an appropriate method for extracting target flavonoids.

## 4. Conclusion

This examination has exhibited another technique for distinguishing and measuring the dynamic constituents in *P*. *acacia*. This method offered many usefulness such as shorter scientific time, less reagents utilization, and straightforwardness over existing frameworks.

## Figures and Tables

**Table 1:** Analytical parameters of the MSPD-HPLC method.

Analyte	Calibration curve	Linear range (*µ*g/mL)	Linearity (*r*2)	LOD (*µ*g/mL)	LOQ (*µ*g/mL)	Intraday precision (RSD%) (*n* = 6)	Interday precision (RSD%) (*n* = 6)
RU	*y* = 54596*x* − 1773.8	5–160	0.9997	4.56	13.82	1.29	1.65
QU	*y* = 53962*x* − 28420	5–160	0.9998	3.97	12.05	1.68	2.38
CA	*y* = 53686*x* − 81042	5–160	0.9998	4.19	12.70	0.97	2.16
KA	*y* = 54358*x* − 47122	5–160	0.9996	5.07	15.39	1.52	2.47

**Table 2:** Recovery studies for the determination of four flavonoids from samples with known concentration (*n* = 3).

Analyte	Addition (*µ*g/mL)	Recovery (%) (mean ± SD)	RSD (%)
RU	30	99.49 ± 1.32	1.33
	60	98.14 ± 1.43	1.45
	120	96.87 ± 1.20	1.24

QU	30	98.44 ± 4.38	4.45
	60	98.71 ± 1.24	1.26
	120	97.98 ± 2.93	2.99

CA	30	97.83 ± 3.92	4.01
	60	99.04 ± 2.04	2.06
	120	98.21 ± 1.33	1.36

KA	30	98.37 ± 1.25	1.27
	60	97.66 ± 1.54	1.57
	120	100.54 ± 1.35	1.34

**Table 3:** Comparison of MSPD with other extraction methods for the extraction of main flavonoids in *P*. *acacia*.

	MSPD	Soxhlet	Sonication
Total extraction yield (mean ± SD, mg/g)			
Sample (gm)	0.1	0.5	0.25
Solvent	8 mL MeOH	100 mL MeOH	40 mL MeOH
Time	15 min	6.5 h	0.5 h
Special apparatus	NO	Soxhlet	Ultrasonicator

## Data Availability

All the data related to these findings are included in the manuscript.
